# Retraction Note: A novel necroptosis signature for predicting survival in lung adenocarcinoma

**DOI:** 10.1186/s12920-024-02030-2

**Published:** 2024-10-16

**Authors:** Kui Zang, Min Wang, Xingxing Zhu, Bin Yao, Ying Huang

**Affiliations:** Department of ICU, the Affiliated Huaian No.1 People’s Hospital of Nanjing Medical University, Jiangsu Province, Huai’an, No.1, Huanghe West Road, Huaiyin District 223300 China

**Retraction Note: BMC Medical Genomics (2023) 16:305**

10.1186/s12920-023-01748-9

The Editor has retracted this article after its authorship could not be verified. Additional concerns were raised around the ethics declaration as it is unclear how consent was obtained for a retrospective dataset-based study. The authors did not respond to requests for clarification. Ying Huang has stated that he was not an author on this article. The authors did not respond to correspondence from the Editor about this retraction.

